# Factors associated with HIV-1 resistance to integrase strand transfer inhibitors in Spain: Implications for dolutegravir-containing regimens

**DOI:** 10.3389/fmicb.2022.1051096

**Published:** 2022-12-12

**Authors:** Horacio Gil, Elena Delgado, Sonia Benito, María Moreno-Lorenzo, Michael M. Thomson, the Spanish Group for the Study of Antiretroviral Drug Resistance

**Affiliations:** HIV Biology and Variability Unit, Centro Nacional de Microbiología, Instituto de Salud Carlos III, Madrid, Spain

**Keywords:** HIV-1, Spain, integrase strand transfer inhibitors, resistance mutation, antirretroviral resistance

## Abstract

Integrase strand transfer inhibitor (INSTI)-containing regimens in HIV-1-infected patients have experienced a global increase. Recently, WHO has emphasized the need to fast-track the transition to dolutegravir (DTG)-based antiretroviral (ARV) treatments. However, continued surveillance of INSTI resistance is recommended. In this study, clinical, epidemiological, and virological features associated with INSTI resistance diagnosed in Spain were analyzed. Samples collected between 2008 and 2021 from HIV-1-infected patients were analyzed in integrase, protease, and reverse transcriptase using Sanger population sequencing. ARV drug resistance was evaluated with the Stanford University HIVdb program. Among 2,696 patients, 174 (6.5%) had INSTI resistance, all of them to first-generation INSTIs, and 71 (2.6%) had also resistance to second-generation INSTIs. Of these, only 5 individuals were exposed to DTG as the only INSTI, in whom resistance development was associated with poor treatment adherence and/or resistance to other ARV classes. Of newly HIV-1-diagnosed individuals, 0.92% harbored INSTI-resistant viruses, with low prevalences maintained along time, and only one had low-level resistance to DTG. Persons who inject drugs, age over 39 years, resistance to other ARV classes, and longer time from diagnosis were associated with INSTI resistance (*p* < 0.001). Non-subtype B INSTI-resistant viruses lacked the Q148H + G140S resistance pathway and showed lower INSTI resistance levels than subtype B viruses. In conclusion, INSTI resistance is uncommon and associated with long-term infections, older age and additional resistance to other ARV drug classes, and is rare in newly diagnosed HIV-1 infections. Our results also support the preferential use of DTG-containing regimens in first-line treatments, although surveillance of INSTI resistance is encouraged.

## Introduction

Integrase strand transfer inhibitors (INSTIs) are a family of antiretrovirals (ARVs) for the treatment of HIV-1 infections. Raltegravir (RAL) and elvitegravir (EVG) are the approved first-generation INSTIs, which are safe and effective for the treatment of ARV-naïve and -experienced patients (DeJesus et al., 2006; Steigbigel et al., 2008; Lennox et al., 2009). While these drugs induce a strong inhibition of HIV-1 replication, they have a modest genetic barrier to resistance, selecting for INSTI resistance mutations that reduce susceptibility to both RAL and EVG (Anstett et al., 2017).

Second-generation INSTIs comprise dolutegravir (DTG) and bictegravir (BIC), which have shown strong suppression of HIV-1 replication, a good safety, high genetic barriers preventing the emergence of drug resistance in large clinical trials (Walmsley et al., 2015; Gallant et al., 2017; Sax et al., 2017; Acosta et al., 2019), and efficacy in treatment-experienced patients (Eron et al., 2013; Castagna et al., 2018).

In the last years, a global increase in the use of INSTI-containing regimens is occurring. In spite of this, very low prevalences of transmitted drug resistance (TDR) to INSTIs have been reported (Inzaule et al., 2018; Casadellá et al., 2020), supporting the use of this drug class in first-line ARV treatments. However, the great increase in the use of second-generation INSTIs and cross-resistance among INSTIs advocate for a continued surveillance of TDR, as well as monitoring their clinical impact.

WHO has proposed DTG as a component of first- and second-line ARV treatments (WHO, 2018) and more recently has emphasized the need to fast-track the transition to DTG-based ARV treatments due to the high prevalence of resistance to non-nucleoside reverse transcriptase inhibitors (NNRTI) in pretreated patients found in some countries (WHO, 2021).

In this study, we analyze the clinical, epidemiological, and virological features of the patients with INSTI resistance included in our database from 2008 to 2021 and the emergence and trends of INSTI resistance. We also focus on DTG to evaluate potential consequences of the widespread inclusion of this drug in first- and second-line ARV drug regimens.

## Materials and methods

### Patients

Samples from individuals diagnosed of HIV-1 infection in Spain which were sent to the HIV Biology and Variability Unit, at Centro Nacional de Microbiología, Instituto de Salud Carlos III (CNM-ISCIII), for INSTI resistance analysis during 2008–2021 were included in the study.

### Nucleic acid extraction, amplification, and sequencing

Nucleic acids were extracted from plasma or whole blood samples. RNA was extracted from 1 ml plasma using NUCLISENS® easyMAG® (BioMérieux, Marcy l’Etoile, France) and DNA was extracted from 200 μl whole blood using QIAamp® DNA DSP blood mini kit (Qiagen, Hilden, Germany), following the manufacturer’s instructions. An integrase-coding fragment of *pol* (HXB2 positions 4,160–5,220) was amplified by RT-PCR followed by nested PCR from RNA, or by nested PCR from DNA. Reagents, PCR thermal profiles, and primers are described in Supplementary Tables 1, 2. The protease-reverse transcriptase (Pr-RT) fragment was amplified as previously described (Gil et al., 2022).

Population sequencing was performed with ABI Prism BigDye Terminator Cycle Sequencing kit and ABI 3730 XL sequencer (Applied Biosystems, Foster City, CA, U.S.A.) at the Genomic Unit of Instituto de Salud Carlos III. Sequences were assembled with SeqMan Pro v.12.2.1 (DNA STAR Lasergene, Madison, WI, USA) and edited with BioEdit v.7.2.5 (Hall, 1999).

### Integrase strand transfer inhibitor resistance analysis

The obtained sequences were analyzed with the Stanford University HIV Drug Resistance Database HIVdb program (version 8.9-1^1^) for the detection of drug resistance (Rhee et al., 2003; Shafer, 2006). Sequences predicted to have a potential low-level resistance to INSTIs were considered susceptible for the objectives of this study. Sequences containing drug resistance mutations (DRMs) associated with apolipoprotein B mRNA editing enzyme, catalytic polypeptide (APOBEC) activity, as determined by the HIVdb program, were excluded from further analysis. A single sequence per patient was considered. In the cases where several sequences were available from a patient with INSTI resistance, the one sampled closer to HIV-1 diagnosis showing INSTI resistance was selected.

Codon usage bias in subtype B and non-subtype B HIV-1 infections was analyzed calculating the frequencies of each triplet in our sequence set and the cost of each mutation using the values described previously (Theys et al., 2019).

### Phylogenetic analyses and genetic form classification

Sequences were analyzed phylogenetically by an approximately maximum-likelihood method using FastTree2 (Price et al., 2010). In these analyses, the general time reversible model of nucleotide substitution with CAT approximation to account for among-site heterogeneity in substitution rates (GTR + CAT) was used, and the reliability of nodes was assessed with Shimodaira-Hasegawa (SH)-like local support values. Classification of sequences in subtypes and circulating recombinant forms was based on clustering with clade references in these trees. Sequences suspected of intersubtype recombination were subsequently analyzed by bootscanning with SimPlot v3.5 (Lole et al., 1999).

### Statistical analysis

Epidemiological factors associated with INSTI resistance were analyzed with chi-squared, Fisher’s exact, and Mann–Whitney tests. The frequencies of DRMs and resistance levels to the different INSTIs were compared between subtype B and non-subtype B viruses with chi-squared and Fisher’s exact tests. The analyses were performed using the STATA statistical software package version 17 (Stata Corporation, College Station, TX, US). Associations were considered statistically significant at a value of *p* < 0.05.

### Ethics statement

Sequences derive from ARV resistance genotypic tests. The use of anonymized, de-identified clinical/demographic and sequence data was reviewed and approved under an exempt protocol by the Bioethics and Animal Well-being Committee of Instituto de Salud Carlos III, with report numbers CEI PI 38_2016-v3 (dated 20 June 2016) and CEI PI 31_2019-v5 (dated 6 November 2019). This study did not require written informed consent by study participants, except for those who donated samples different from the ones obtained in clinical practice.

## Results

### Frequency of INSTI resistance and associated factors

Resistance mutations in integrase conferring, at least, low level of resistance to any INSTI were detected in 174 (6.5%) of 2,696 patients analyzed. A comparison between both populations, with and without INSTI resistance, is shown in Table 1. Persons who inject drugs (PWID) were the main transmission category in the group with INSTI resistance (37% vs. 19% in those without INSTI resistance). The main age groups with INSTI resistance were 40–49 and ≥ 50, with similar percentages in both age groups; joining them together (ages 40 and older), percentages were 73% vs. 56% in the groups with and without INSTI resistance, respectively. The average time from diagnosis was double in patients with INSTI resistance (14.4 years vs. 7.2 years). Differences between groups with and without INSTI resistance with regard to transmission routes, age, and time from HIV-1 diagnosis were statistically significant (*p* < 0.001; Table 1).

**Table 1:** Characteristics of the patients with INSTI resistance included in the study.

			INSTI resistance^*^				
			No		Yes		
Variable	Categories	Total patients	*N*	%	*N*	%	Value of *p*
*Gender*	Female	595	550	22	45	27	0.30
	Male	2042	1918	77	124	73	
	Transsexual	10	10	0.40			
	No data	49	44		5		
Transmission route**	Heterosexual	606	565	35	41	33	<0.0001
	MSM	511	490	30	21	17	
	MNSST	208	197	12	11	8.8	
	PWID	347	301	19	46	37	
	Vertical	46	43	2.6	3	2.4	
	Others	28	20	1.2	2	1.6	
	No data	956	906		50		
Region of origin	Spain	1,678	1,570	75	108	72	0.08
	Latin America	299	279	13	20	13	
	Sub-Saharan Africa	143	126	6.0	17	11	
	North Africa	31	28	1.3	3	2.0	
	Europe	64	62	3.0	2	1.3	
	Others	24	24	1.1			
	No data	457	433		24		
Age group	≤29	464	447	18	17	10	<0.001
	30–39	673	643	26	30	18	
	40–49	774	710	28	64	37	
	≤50	756	696	28	60	35	
	No data	29	26		3		
Other resistance^†^	PI	141	110	5.0	31	22	<0.0001
	NRTI	345	254	11	91	64	<0.0001
	NNRTI	500	438	20	62	53	<0.0001
	Pr-RT sequenced	2,357	2,214		143		
Time from diagnosis^‡^		7.7	7.2		14.4		<0.0001
Total		2,696	2,522		174	6.5	

^*^Percentages were calculated over the total of patients with data.^**^Transmission route: MSM: men who have sex with men; MNSST: men with non-specified sexual transmission; PWID: persons who inject drugs.^†^Resistance to other antiretrovirals. Pr-RT sequenced: number of patients with Pr-RT sequence available; PI: protease inhibitors; NRTI: nucleoside reverse transcriptase inhibitors; NNRTI: non-nucleoside reverse transcriptase inhibitors.^‡^Average years from HIV-1 diagnosis.

Patients with INSTI resistance also had a higher percentage of DRMs in Pr-RT, compared to the group without INSTI resistance, associated with resistance to protease inhibitors (PI; 22% vs. 5%), to nucleoside reverse transcriptase inhibitors (NRTI; 64% vs. 11%), and to NNRTI (53% vs. 20%), with statistically significant associations for all three drug classes (*p* < 0.0001; Table 1).

Most (75%) viruses in the studied population were of subtype B. There was no statistically significant difference in the frequency of INSTI resistance between subtype B (6.5%) and non-subtype B (6.2%) viruses. Among non-subtype B viruses, the most frequent genetic form was CRF02_AG, which represented 33% (14/42) of non-B viruses with INSTI resistance, and within which the prevalence of INSTI resistance was 8.1%. The highest prevalence of INSTI resistance was found in CRF89_BF (67%), although only 6 patients were infected with viruses of this genetic form (Table 2).

**Table 2:** Frequency of INSTI resistance among HIV-1 genetic forms.

	All patients			Newly-diagnosed patients		
		With INSTI resistance		With INSTI resistance
Genetic form	*N*	*N*	%	*N*	*N*	%
B	2016	132	6.5	642	4	0.62
Non-B	680	42	6.2	332	5	1.5
A	64	2	3.1	36	1	2.8
C	45	1	2.2	25		
D	8	1	13	4		
F	137	5	3.6	82	2	2.4
G	50	4	8.0	23	1	4.3
CRF02_AG	172	14	8.1	73	1	1.4
CRF47_BF	22	2	9.1	13		
CRF89_BF	6	4	67	2		
Other CRFs	75	3	4.0	33		
URFs	96	5	5.2	39		
Others	6	0	0.0	2		
Total	2,696	174	6.5	978	9	0.92

The most frequent INSTI in the treatment regimens of patients with INSTI resistance was RAL, used in 58%, followed by EVG, used in 7.5%, and DTG, used in 4.6%. The remaining 30% were patients exposed to two different INSTIs or an unspecified INSTI.

### INSTI resistance in newly diagnosed and INSTI-naïve patients

**Figure 1:**
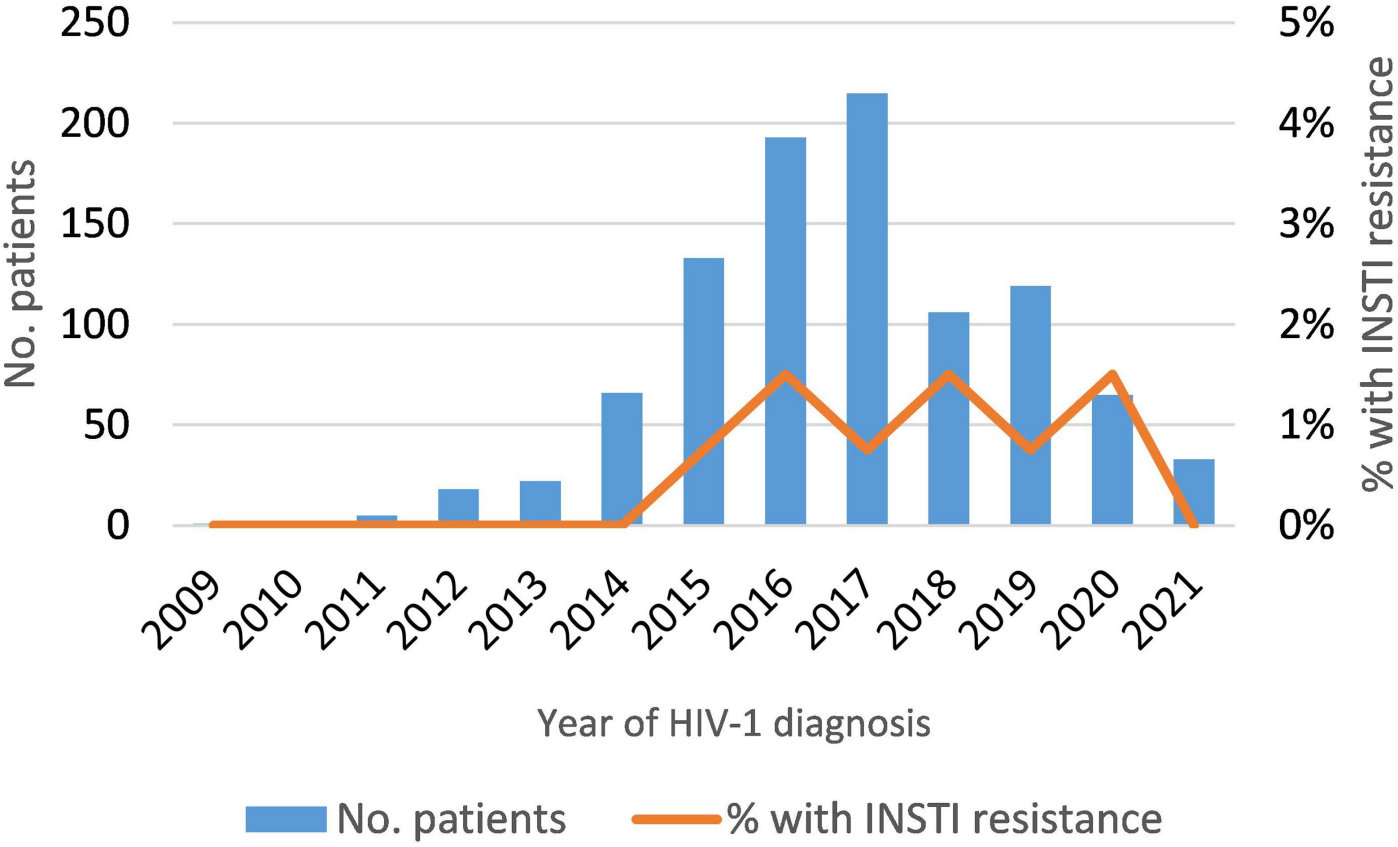
Temporal trend of INSTI resistance in newly-diagnosed HIV-1 patients analyzed in this study (2009–2021).

Among newly-diagnosed patients (NDs), the prevalence of INSTI resistance was 0.92% (9/978). It was lower in subtype B (0.6%) than in non-subtype B (1.5%) infections (Table 2), but the difference was not statistically significant. The 5 non-subtype B viruses with INSTI resistance belonged to subtypes A, F (two patients), and G, and to CRF02_AG (Table 2). The annual prevalence of INSTI resistance among NDs was in the range of 0.75–1.5%, showing a stable trend in 2015–2020 (Figure 1). In 2021, no patients with INSTI resistance were detected, although only 33 patients were analyzed that year.

Among 174 patients with INSTI resistance, only 25 (14%) were INSTI-naïve, 11 of whom were infected with non-subtype B and 14 with subtype B strains (Table 3). Among INSTI-naïve patients with INSTI resistance, resistance levels were low in most (88%) of them, with the most common mutation being G163K/R (INSTI accessory mutations), detected in 17 (68%), followed by E138K, detected in 4 (16%). In these patients, resistance to DTG or BIC was found in only one infection, carrying the S230R accessory mutation, which is associated with low-level resistance to these INSTIs (Table 3).

**Table 3:** Predicted resistance level and INSTI mutations identified in INSTI-naïve patients.

Mutations		Genetic form			INSTI resistance level^†^			
Major	Accessory	All (*n* = 25)	*B* *n* = 14)	Non-B (*n* = 11)	RAL	EVG	DTG	BIC
	G163R	11^‡^	5^‡^	6^‡^	Low	Low	Susceptible	Susceptible
	G163K	6	3	3^‡^	Low	Low	Susceptible	Susceptible
E138K		4^‡^	2^‡^	2^‡^	Low	Low	Susceptible	Susceptible
	S230R	1	1		Low	Low	Low	Low
E92G		1^‡^	1^‡^		Low	Intermediate	Susceptible	Susceptible
	V151A	1	1		Low	Intermediate	Susceptible	Susceptible
N155H	T97A	1	1		High	High	Susceptible	Susceptible

^†^RAL: Raltegravir, EVG: Elvitegravir, DTG: Dolutegravir, BIC: Bictegravir.^‡^Newly-diagnosed patients: G163R was found in 3 NDs (1 subtype B and 2 non-subtype B). E138K was found in 2 ND (1 subtype B and 2 non-subtype B).

### Resistance levels to INSTIs

All INSTI-resistant viruses exhibited, at least, a predicted low-level resistance to RAL, with 70, 66, and 12% showing high-level resistance to RAL, EVG, and both DTG and BIC, respectively (Table 4). We also compared resistance levels to different INSTIs. Regarding EVG, resistance levels to this INSTI were similar to those observed to RAL, although 3.5% (6/174) RAL-resistant viruses were susceptible to EVG and 15% (8/52) viruses with low or intermediate resistance to RAL had high-level resistance to EVG, which was associated with T66A/I and E92Q mutations. Interestingly, 59 and 61% of RAL-resistant viruses were susceptible to DTG and BIC, respectively, and only 14% (6/43) viruses with low-level RAL resistance showed intermediate resistance to these second-generation INSTIs (Table 4), due to R263K mutation.

**Table 4:** Resistance levels to raltegravir compared to the other INSTIs.

		Predicted resistance level to raltegravir									
		Low		Intermediate		High		Total	
INSTI	Resistance level	*N*	%	*N*	%	*N*	%	*N*	%
Elvitegravir	Susceptible	0		0		6	4.9	6	3.5
	Low	33	77	0		4	3.8	37	21
	Intermediate	8	19	3	33	5	4.1	16	9.2
	High	2	4.7	6	67	107	88	115	66
Dolutegravir	Susceptible	34	79	8	89	61	50	103	59
	Low	3	7.0	0		9	7.4	12	6.9
	Intermediate	6	14	1	11	31	25	38	22
	High	0		0		21	17	21	12
Bictegravir	Susceptible	37	86	8	89	61	50	106	61
	Low	0		0		12	9.8	12	6.9
	Intermediate	6	14	1	11	28	23	35	20
	High	0		0		21	17	21	12
Total		43	24.7	9	5.2	122	70	174	100

### INSTI resistance levels in subtype B and non-subtype B viruses

**Figure 2:**
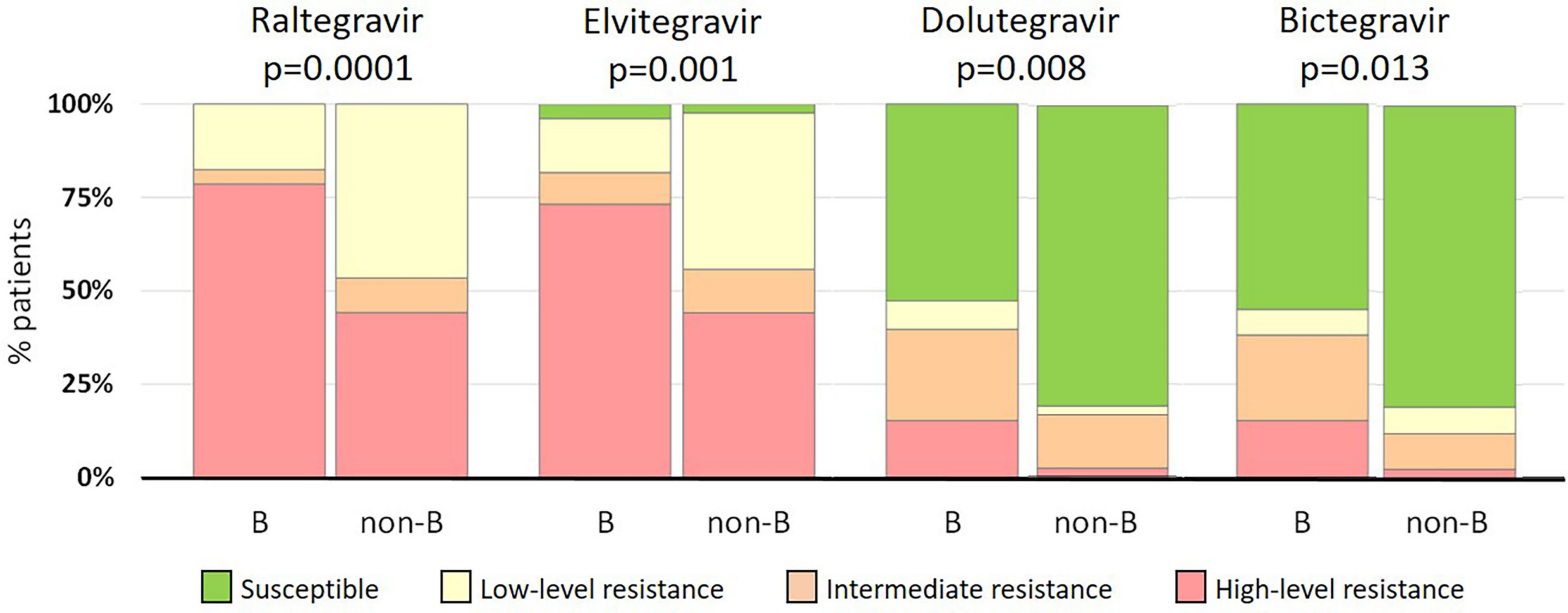
Comparison of INSTI resistance levels between subtype B (*n* = 132) and non-subtype B (*n* = 42) infections.

Despite the lack of statistical differences in the frequency of INSTI-resistant viruses between patients infected with B and non-B strains, non-subtype B strains exhibited lower resistance levels than subtype B strains (Figure 2). High-level resistance to RAL was found in 79% (104/132) and 43% (18/42) of subtype B and non-subtype B infections, respectively. Similarly, high-level resistance to EVG was found in 73% (97/132) and 43% (18/42) of the respective categories. Interestingly, of non-B strains resistant to first-generation INSTIs, 81% (34/42) were susceptible to both second-generation INSTIs, a proportion that was higher than in subtype B strains, in which 52% (69/132) and 55% (72/132) viruses resistant to first-generation INSTIs where susceptible to DTG and BIC, respectively. Differences in INSTI resistance levels between B and non-B strains were statistically significant, ranging from *p* = 0.0001 for RAL to *p* = 0.013 for BIC (Figure 2).

### INSTI resistance mutations

The most frequent major INSTI DRM was N155H, which was found in 35% INSTI-resistant viruses, followed by G140S, Q148H, and Y143X, found in 18, 17, and 12%, respectively. Regarding the accessory DRMs, T97A was detected in 15% of the patients, followed by G163R, G163K, and D232N, detected in 14, 7.5, and 6.3%, respectively. The most frequent INSTI DRM combination was Q148H + G140S, which was observed in 16% of INSTI-resistant viruses, with the additional presence of T97A in 8 of them. G118R mutation, associated with DTG resistance, was not found in any patient. The frequencies of all INSTI DRMs are shown in Table 5.

**Table 5:** Drug resistant mutations found in subtype B and non-subtype B infections.

Type	Mutation	All (*n* = 174)		Subtype B (*n* = 132)		Non-subtype B (*n* = 42)		Value of *p*
		*N*	%	*N*	%	*N*	%	
Major	T66A/I/K	6	3.5	4	3.0	2	4.8	0.60
	E92Q/G	13	7.5	8	6.1	5	12	0.24
	E138A/K/T	18	10	16	12	2	4.8	0.18
	G140S	32	18	32	24			**<0.0001**
	G140A	7	4.0	6	5.0	1	2.4	0.61
	Y143X	21	12	15	11	6	14	0.61
	S147G	7	4.0	6	5.0	1	2.4	0.61
	Q148H	29	17	29	22			**0.0001**
	Q148R	18	10	17	13	1	2.4	**0.044**
	Q148K	1	0.57	1	0.76			0.76
	N155H	60	35	49	37	11	26	0.27
	N155S	1	0.57	1	0.76			0.76
	R263K	7	4.0	4	3.0	3	7.1	0.45
Accessory	H51Y	2	1.1	2	1.5			0.57
	Q95K	2	1.1			2	4.8	0.11
	T97A	26	15	23	17	3	7.1	0.16
	P142T	1	0.57	1	0.76			0.76
	G149A	1	0.57	1	0.76			0.76
	V151A	1	0.57	1	0.76			0.76
	E157Q	6	3.5	3	2.3	3	7.1	0.31
	G163R	24	14	13	9.8	11	26	**0.009**
	G163K	13	7.5	6	5.0	7	17	**0.019**
	S230R	7	4.0	5	3.8	2	4.8	0.54
	D232N	11	6.3	11	8.3			**0.043**
Double^*^	Q148H + G140S	27	16	27	20			**0.0002**
	Q148R + G140S/A	10	5.7	9	6.8	1	2.4	0.31
	Q148R + E138K	6	3.4	6	5.0			0.37
	N155H + D232N	11	6.3	11	8.3			**0.043**
	N155H + T97A	10	5.7	9	6.8	1	2.4	0.31
Triple^*^	Q148H + G140S + T97A	8	4.6	8	6.1			0.10

Statistically significant p-values are in bold type. ^*^Only combinations found in 5 or more patients are included.

Different DRM patterns were observed in subtype B and non-subtype B viruses. Among major DRMs, G140S (*p* < 0.0001), Q148H (*p* = 0.0001), and Q148R (*p* = 0.044) were found mainly or exclusively in subtype B-infected patients (Table 5). Similarly, the accessory DRM D232N (*p* = 0.043) was associated with subtype B infections, while G163R (*p* = 0.009) and G163K (*p* = 0.019) were detected mainly in non-subtype B infections (Table 5; Supplementary Figure 1). G163K/R was frequent among F subtype and BF recombinant viruses, as 67% of non-B viruses with these mutations were of subtype F in integrase, and 4 of 6 CRF89_BF INSTI-resistant viruses carried G163R.

### Codon usage among subtype B and non-subtype B HIV-1 strains

Large differences in the frequency of codon usage were observed between subtype B and non-subtype B strains in amino acids E92 (GAG, 64% vs. 5.7%; and GAA, 29% vs. 92%), G140 (GGC, 72% vs. 7.8%; and GGA, 3.6% vs. 74%), G163 (GGA, 80% vs. 47%; and GGG, 8% vs. 41%), and D232 (GAT, 78% vs. 22%; and GAC, 11% vs. 73%) (Supplementary Table 3). However, only for G140 there was a difference in the substitution cost of change from the most frequent codon to the resistance-associated amino acid (G140S), which was higher for the non-subtype B strains (GGA, score 7.4) than for subtype B viruses (GGC, score 1) (Supplementary Table 3).

Regarding the R263K and G118R mutations, which are associated with DTG resistance, there were no differences in codon usage, although the substitution cost was higher for G118R (score 5.8) than for R263K (score 1; Supplementary Table 3).

### INSTI resistance in patients exposed only to DTG

In 8 (4.6%) of the 174 patients with INSTI resistance, DTG was the only INSTI used in the treatment regimen. Three of them only had G163R/K, which keep viruses susceptible to DTG, while 5 showed intermediate (*n* = 3) or low (*n* = 2) level resistance to DTG, associated with R263K and S230R mutations, respectively (Table 6). All patients with intermediate-level resistance to DTG had DRMs to NNRTI and NRTI, which were M184V and K103N in two of them (Table 6). Four of 5 patients with DTG resistance had poor therapeutic adherence (as assessed by their physicians when the sample was sent for resistance testing) and in the fifth, the treatment regimen had been changed after renal failure (Table 6).

**Table 6:** Patients with INSTI resistance exposed to dolutegravir as the only INSTI.

						Antiretroviral therapy^†^			Drug resistance mutations^‡^				
Gender	Age	Transmision route*	Geographical origin	Years from diagnosis	Genetic form	Current	Previous	Adherence	PI	NRTI	NNRTI	INSTI	Resistance level to DTG
F	18	Vertical	Sub-Saharan Africa	18	CRF02_AG	DTG + ABC + 3TC	EFV + ABC + 3TC	Poor	No	L74V	K103N, V179E	R263K	Intermediate
M	31	ND	ND	<1	CRF02_AG	TDF + EFV + 3TC	DTG + ABC + 3TC	Poor	No	K70E, L74V, M184V	K103N, G190A	E157Q, R263K	Intermediate
M	20	MSM	Latin America	<1	B	DTG + ABC + 3TC^ƪ^	3TC + TDF + DRV/r	Good	No	M184V	No	R263K	Intermediate
M	56	PWID	Spain	31	B	DTG + ABC + 3TC	Multiple	Poor	No	No	No	S230R	Low-Level
F	53	ND	ND	31	B	DTG + 3TC	ABC + 3TC + ETR	Poor	No	No	E138A	S230R	Low-Level
F	65	ND	ND	7	D	TDF + FTC + DTG	None	Poor	No	No	No	G163R	Susceptible
M	52	HET	Sub-Saharan Africa	12	CRF02_AG	DTG + ABC + 3TC	ABC + 3TC + EFV	Poor	NA	NA	NA	G163K	Susceptible
M	26	HSH	Latin America	<1	B	TDF + FTC + DTG	None	Good	No	No	No	G163R	Susceptible

^*^PWID, person who injects drugs; MSM, man who has sex with men; HET, heterosexual. ND, no data.^†^DTG, Dolutegravir; ABC, Abacavir; 3TC, Lamivudine; FTC, Emtricitabine; TDF, Tenofovir; ETR, Etravirine; EFV, Efavirenz; DRV/r, Danuravir/ritonavir.^‡^Drug classes to which resistance was found: PI, protease inhibitors; NRTIs, nucleoside reverse transcriptase inhibitors; NNRTI, non-nucleoside reverse transcriptase inhibitors; INSTI, integrase strand transfer inhibitors. No, no mutations. NA, no sequence available.^ƪ^Change in the treatment due to renal failure.

## Discussion

In this study we have analyzed epidemiological and virological features associated with INSTI resistance in our cohort of 2,696 patients, including 25% infected with non-subtype B strains. INSTI resistance was associated with PWID, patients 40 years of age or older, and long-term infections. PWID frequently have difficulties to fit antiretroviral treatment in their daily routine, increasing their risk for reduced adherence (Lert and Kazatchkine, 2007; Vervoort et al., 2007). Other factors, like focusing in acquisition of illegal drugs, interactions of these drugs with treatments that induce side effects, homelessness, or difficulties in obtaining medication, have been also associated with reduced adherence in PWID (Witteveen and van Ameijden, 2002; Vervoort et al., 2007). Close follow-up and new strategies to improve adherence in HIV-1-infected PWID are necessary to prevent emergence of INSTI resistance in this population.

Accumulation of DRMs to PI, NRTI, and NNRTI was also associated with INSTI resistance. These DRMs can reduce the activity of ARVs used in combination treatments with INSTI, increasing the risk of a functional INSTI monotherapy and explaining the emergence of INSTI resistance (Cahn et al., 2013; Naeger et al., 2016; Modica et al., 2019; Underwood et al., 2022). Also, the presence of DRMs in Pr-RT could indicate a reduced adherence of the patient to the treatment, which can enhance the appearance of INSTI resistance mutations (Nachega et al., 2011; von Wyl et al., 2013; Acosta et al., 2022).

Transmitted INSTI resistance is currently a rare event, which we found only in 0.92% of the 978 NDs, with a stable trend in the last years (2015–2020). Moreover, the most frequent INSTI resistance mutation found among these patients was the polymorphic G163K/R mutation (Tzou et al., 2020), which is only associated with a low-level resistance to RAL and EVG. The low frequency of transmitted INSTIs resistance mutations has been reported in previous surveillance studies and meta-analyses (Doyle et al., 2015; Hauser et al., 2018; Inzaule et al., 2018; Raffaelli et al., 2018; Alvarez et al., 2019; Liu et al., 2019; Casadellá et al., 2020; Mbisa et al., 2020; Tzou et al., 2020; Semengue et al., 2021). This scenario supports the use of INSTIs in first-line therapeutic regimens. However, continued surveillance of ARV resistance is strongly recommended (WHO, 2014), as increase in the prevalence of resistance to INSTI could occur with the growing use of these drugs. Therefore, updated surveillance data will allow choosing the most appropriate treatment strategy at the global and national levels.

The three main RAL resistance pathways, N155H, Q148H/R/K, and Y143C/R (Cooper et al., 2008; Anstett et al., 2017), the latter at low frequency, were found among INSTI-resistant viruses in our study. N155H and Q148X affect virus fitness, to a lesser extent in the case of N155H. Thus, under RAL treatment, N155H emerged earlier than Q148X (Hu and Kuritzkes, 2010). Such difference in fitness could explain the high frequency of N155H found in our study, where INSTI-resistant viruses were mainly exposed to RAL. Fitness loss can be compensated by secondary mutations, which appear sequentially, inducing higher levels of resistance to RAL (Hu and Kuritzkes, 2010; Anstett et al., 2017). This can explain the frequent finding of Q148H/R + G140S/A, Q148R + E138K, and N155H + T97A combinations in our study. Moreover, viruses with Q148H + G140S, which was the most frequent combination in our cohort, are fitter than viruses with single mutations or double mutants with N155H, and therefore can be preferably selected along time under RAL pressure (Hu and Kuritzkes, 2010). Later, along the evolution of drug resistance, other mutations can emerge, such as T97A, which we have found frequently with the Q148H + G140S combination.

The most common INSTI resistance mutations in persons with virological failure under DTG-containing regimens were R263K, G118R, N155H, and Q148H/R (Eron et al., 2013; Castagna et al., 2018; Rhee et al., 2019), with R263K and G118R being predominant in INSTI-naïve patients (Cahn et al., 2013, 2022; Underwood et al., 2022; Vavro et al., 2022). However, G118R was not found in our cohort, probably due to the preferential codon usage in this position, GGC/GGT, whose change to arginine has a high substitution cost compared to the rare GGA/GGG codons. This bias in codon usage is found in almost all HIV-1 subtypes (Brenner et al., 2016; Theys et al., 2019) and represents an additional genetic barrier to the development of DTG resistance. Indeed, the presence of these rare triplets has been postulated to be a requirement for the development of G118R mutation (Brenner et al., 2016).

The frequency of INSTI resistance in subtype B and non-subtype B infections was similar, although the latter group showed lower resistance levels to all INSTIs. This is mainly due to the different INSTI mutation patterns observed, with the Q148H + G140S combination, frequent in subtype B, being absent among non-subtype B infections, similarly to other observational studies (Brenner et al., 2011; Doyle et al., 2015; Fourati et al., 2015; Hauser et al., 2018; Raffaelli et al., 2018; Modica et al., 2019; Sánchez et al., 2020; Scutari et al., 2020; Tzou et al., 2020). This bias is related to the different codon usage in G140, which has a higher substitution cost for acquiring the G140S resistance mutation in non-subtype B strains (Theys et al., 2019). In addition, the G163K/R polymorphic mutations were associated to subtype F and BF recombinant viruses, and D232N in combination with N155H was associated to subtype B, similarly to the findings in other studies (Sánchez et al., 2020; Tzou et al., 2020).

DTG has a high genetic barrier (Kobayashi et al., 2011; Quashie et al., 2012; Rhee et al., 2019), which seems to be higher for non-subtype B strains, according to the absence in them of the Q148H + G140S resistance pathway found in this and previous studies. (Brenner et al., 2011; Doyle et al., 2015; Fourati et al., 2015; Modica et al., 2019; Sánchez et al., 2020; Scutari et al., 2020; Tzou et al., 2020). Non-subtype B strains are predominant in most low- and middle-income countries (Hemelaar et al., 2019), which is an additional advantage for the use of DTG in these countries, where genotyping testing capacity for identification of DRMs that can jeopardize DTG-based therapies is frequently less available.

DTG remains a therapeutic option in most infections with INSTI resistance in our cohort, among which 59% are still susceptible to DTG. In fact, DTG 50 mg twice daily, instead of the usual once daily regimen, with an optimized background therapy has been shown to be efficient in the VIKING and PRESTIGIO trials, in which patients with failing regimens and different INSTI resistance levels were enrolled (Eron et al., 2013; Castagna et al., 2018), although patients with Q148X mutations showed a reduced response to the therapy (Castagna et al., 2014).

Virological failure in INSTI-naïve patients who are prescribed DTG-containing regimens is rare and has been associated with low adherence, DTG monotherapy, previous presence of resistance mutations in Pr-RT, and the interaction of INSTI with other drugs, which reduce the effective DTG plasma concentration (Cahn et al., 2013; Cevik et al., 2020; Cahn et al., 2022; Revollo et al., 2022; Underwood et al., 2022; Vavro et al., 2022). These patients were also rare in our cohort, where only 5 individuals exposed to DTG as the only INSTI developed mutations associated to intermediate- or low-level resistance to DTG. The emergence of such mutations could be related to low therapeutic adherence and the presence of previous drug resistance mutations in Pr-RT. Indeed, the presence of M184V and K103N could have contributed to the development of DTG resistance in two of them.

As a limitation, our cohort includes a greater representation of patients with INSTI resistance compared to the HIV-1-infected population, due to a higher representation of patients with therapeutic failure in the samples received in our laboratory for ARV resistance testing. However, this bias allows us to have a higher number of samples for analysis of INSTI resistance mutations and their associated features.

INSTI resistance levels determined in our study were based on the Stanford HIVdb algorithm, which uses phenotypic data to assign a value to each mutation to predict the resistance level of the virus. However, since there are fewer phenotypic studies with non-subtype B strains than with subtype B viruses, the contributions of some mutations to the resistance level of non-subtype B strains may not be well characterized and, therefore, the level of resistance could be underestimated. Additional phenotypic analyses with non-subtype B strains are needed to improve the resistance level predictions with the available algorithms.

In conclusion, in our study, INSTI resistance was uncommon and was associated with long-term infections, additional resistance mutations to other ARV classes, and probably with low therapeutic adherence. The few cases of INSTI resistance mutations in regimens containing DTG and the low level of transmitted resistance to DTG in our cohort supports the WHO recommendation for the use of DTG in first- and second-line treatments. However, the expected increase in the use of DTG in the next years and its use in settings with high prevalence of NRTI and NNRTI resistance, such as low- and middle-income countries, encourage the implementation of surveillance systems to detect the potential emergence and spread of DTG-resistant strains.

## Members of the Spanish Group for the Study of Antirretroviral Drug Resistance

Aragón: Hospital Universitario Miguel Servet: Ana Mª Martínez-Sapiña, Piedad Arazo; Hospital Clínico Universitario Lozano Blesa: Sonia Algarate; Hospital de Barbastro: Marta Lalana Garcés. Basque Country: Hospital Universitario Basurto: Josefa Muñoz, Mª Carmen Nieto, Sofía Ibarra, Estibaliz Ugalde; Hospital Universitario de Cruces: Luis Elorduy, Elena Berciartua, Josune Goikoetxea, Laura Guío, Mª José Blanco; Hospital de Galdakao: Mª José López de Goicoechea, José Mayo; Hospital Universitario Donostia: Carlos Gustavo Cilla, José Antonio Iribarren, Mª Yolanda Salicio, Maitane Aranzamendi, Maialen Ibarguren. Hospital Universitario de Álava: Juan Carlos Gainzarain, Zuriñe Ortiz de Zárate, Miguel Ángel Morán, Ester Sáez de Arana, José Joaquín Portu, Carmen Gómez-González. Cantabria: Hospital Universitario Marqués de Valdecilla: M. Eliecer Cano. Castilla y León: Hospital El Bierzo: Sonia Belén Paredes. Hospital Clínico Universitario de Valladolid: Carmen Hinojosa, Begoña Monteagudo; Hospital Universitario Río Hortega: Belén Lorenzo, Jessica Abadía; Hospital Virgen de la Concha: Teresa Martín-Domínguez, Rosa Martínez-González. Castilla-La Mancha: Hospital Virgen de la Salud: César Gómez-Hernando, José Largo-Pau; Hospital Universitario de Guadalajara: Alejandro González-Praetorius. Hospital Virgen de la Luz: Enrique Prada, Paloma Geijo; Hospital Santa Bárbara: Ana Cosmen; Comunidad Valenciana: Hospital Universitari Sant Joan d’Alacant: Fernando Buñuel, Ana Infante. Extremadura: Hospital de Mérida: Julián Sánchez. Complejo Hospitalario Universitario de Cáceres: Guadalupe Rodríguez. Galicia: Complejo Hospitalario Universitario de Ferrol: Ana Mariño, Patricia Ordóñez, Hortensia Álvarez, Nieves Valcarce, Sabela Sánchez; Complejo Hospitalario Universitario de A Coruña: Ángeles Cañizares, Mª Ángeles Castro, Luz Moldes Suárez. Hospital Universitario Lucus Augusti: Ramón Rabuñal, María José García País, Mª José Gude González, Pilar Alonso, Eva María Romay, Antonio Moreno; Complejo Hospitalario Universitario de Ourense: Juan García Costa, Ricardo Fernández-Rodríguez, Raúl Rodríguez-Pérez, Luis Canoura, María Dolores Díaz-López, Mª Genoveva Naval-Calviño; Complejo Hospitalario Universitario de Vigo: Celia Miralles, Antonio Ocampo, Sonia Pérez-Castro, Jorge Julio Cabrera; Sandra Cortizo Vidal, Guillermo Pousada, Luis Morano; Complejo Hospitalario de Pontevedra: Julio Díz-Arén, Matilde Trigo, Mª Ángeles Pallarés. La Rioja: Hospital San Pedro: José Ramón Blanco, Miriam Blasco. Madrid: Hospital de Fuenlabrada: Julio García Díez, Laura María Molina Esteban, Santiago Prieto-Menchero, Mª Isabel García-Arata; Hospital Clínico San Carlos: Esther Culebras, Iciar Rodríguez-Avial; Hospital Universitario Fundación Jiménez Díaz: Raquel Téllez, Miguel Górgolas, Ángel Luis Castaño, Olalla Calabia, Alfonso Cabello. Hospital Universitario de la Princesa: Laura Cardeñoso. Hospital Universitario Príncipe de Asturias: Juan Cuadros. Fundación Hospital de Alcorcón: Mª José Goyanes, Carolina Campelo. Murcia: Hospital J.M. Morales Meseguer: Pedro Antequera. Navarra: Hospital Universitario de Navarra: Carmen Martín, Carmen Ezpeleta-Baquedano, Aitziber Aguinaga, Irati Arregui García, Ana Miqueleiz, Mª Gracia Ruiz de Alda. Hospital Reina Sofía de Tuleda: Leticia Raquel Armendáriz López, Marta Adelantado Lacasa.

## Data availability statement

The raw data supporting the conclusions of this article will be made available by the authors, without undue reservation.

## Ethics statement

The studies involving human participants were reviewed and approved by Bioethics and Animal Well-being Committee of Instituto de Salud Carlos III. Written informed consent for participation was not required for this study in accordance with the national legislation and the institutional requirements.

## Author contributions

HG, ED, and MT conceived the study and supervised the experimental work. ED and HG performed drug resistance analyses and data curation. HG performed statistical analyses and wrote the manuscript draft, with contributions to the text by MT and ED. SB and MM-L performed experimental work. The members of the Spanish Group for the Study of Antiretroviral Drug Resistance recruited patients and obtained epidemiological and clinical data. All authors read and approved the text.

## Funding

This work was funded through Acción Estratégica en Salud Intramural (AESI), Instituto de Salud Carlos III, projects “Estudios sobre vigilancia epidemiológica molecular del VIH-1 en España,” PI16CIII/00033 and “Epidemiología molecular del VIH-1 en España y su utilidad para investigaciones biológicas y en vacunas”, PI19CIII/0042; and scientific agreements with Consellería de Sanidade, Government of Galicia (MVI 1004/16) and Osakidetza-Servicio Vasco de Salud, Government of Basque Country (MVI 1001/16).

## Conflict of interest

The authors declare that the research was conducted in the absence of any commercial or financial relationships that could be construed as a potential conflict of interest.

## Publisher’s note

All claims expressed in this article are solely those of the authors and do not necessarily represent those of their affiliated organizations, or those of the publisher, the editors and the reviewers. Any product that may be evaluated in this article, or claim that may be made by its manufacturer, is not guaranteed or endorsed by the publisher.

## Footnotes

^1^ http://hivdb.stanford.edu
