# Supplementary material for: Factors associated with HIV-1 resistance to integrase strand transfer inhibitors in Spain: Implications for dolutegravir-containing regimens
Source: Front Microbiol. 2022 Dec 12;13:1051096. doi: 10.3389/fmicb.2022.1051096 (PMC9792149; doi:10.3389/fmicb.2022.1051096)

Supplementary Table 1. Reagents and thermal profile for integrase amplification.

Reagents	RT-PCR (for RNA)	1st PCR (for DNA)	Nested PCR (RNA and DNA)
OneStep RT-PCR kit (QIAGEN) enzyme	1 µl	--	--
GoTaq G2 Hot Start colorless mastermix (Promega)	--	12.5 µl	--
BioTaq DNA polymerase (Bioline)	--	--	0.75 U
RNasin (RNAse inhibitor) (Promega)	5 U	--	-
$MgCl_2$	2.5 mM*	2 mM†	1.8 mM
dNTPs	400 µM	200 µM†	200 µM
Each primer	0.6 µM	0.4 µM	0.4 µM
Reaction volume	25 µl	25 µl	25 µl
Thermal cycling profile			
Reverse transcription	30 min 50ºC	--	--
Hot start activation	15 min 95ºC	--	--
Denaturation	--	2 min 94ºC	2 min 94ºC
Cycling	40 cycles	35 cycles	40 cycles
Denaturation	30 sec 94ºC	30 sec 94ºC	30 sec 94ºC
Annealing	1 min 55ºC	45 sec 55ºC	45 sec 56ºC
Extension	90 sec 72ºC	1 min 72ºC	1 min 72ºC
Final extension	7 min 72ºC	7 min 72ºC	5 min 72ºC

* Included in the 2x kit mastermix; † Included in the 5x kit buffer.

Supplementary Table 2. Primers used in the integrase amplification and sequencing.

Oligo	Use	Direction	HXB2 position	Sequence
RTDS-N-S	1st PCR/RT-PCR	sense	3132-3157	5'- ATA GGG CAG CAT AGA RCA AAA ATA GA
5'HIV-O-A	1st PCR/RT-PCR	antisense	5239-5266	5´- CTC CTG TAT GCA GAC CCC AAT ATG TTG T
INTS	nested PCR/ sequencing	sense	4160-4182	5'- AGC ACA CAA AGG AAT TGG AGG AA
5´HIVNA	nested PCR/ sequencing	antisense	5192-5220	5'- CCC TAG TGG GAT GTG TAC TTC TGA ACT TA
IN-MED-S	sequencing	sense	4647-4671	5'- GGC ATT CCC TAC AAT CCC CAA AGT C
B2-A-N	sequencing	antisense	5043-5067	5'- AAT CAT CAC CTG CCA TCT GTT TTC C

Supplementary Table 3. Codon usage and resistance cost of INSTI resistance mutations in subtype B and non-B subtype strains.

		Subtype B (N=2016)		Non-subtype B (N=680)		Resistance mutations		
**Position**	**Codon**	**N**	**%**	**N**	**%**	**AA***	**Codon†**	**SC‡**
**E92**	**GAA**	591	29	624	92	Q	CAA	5.8
	**GAG**	1281	64	39	5.7	Q	CAG	5.8
	**GAN**	137	6.8	7	1.0			
	**Others**	7	0.3	10	1.5			
**T97**	**ACA**	1928	96	647	95	A	GCA	1
	**ACT**	7	0.35	3	0.44	A	GCT	1
	**ACC**	8	0.40			A	GCC	1
	**ACG**	10	0.50			A	GCG	1
	**ACN**	14	0.69	7	1.0			
	**Others**	49	2.4	23	3.4			
**G118**	**GGA**	24	1.2	11	1.6	R	AGA	1
	**GGT**	136	6.7	86	13	R	CGT	5.8
	**GGC**	1784	88	563	83	R	AGG	4.4
	**GGG**	7	0.35	1	0.15	R	AGG	1
	**GGN**	62	3.1	19	2.8			
	**Others**	3	0.15					
**E138**	**GAA**	1935	96	646	95	K	AAA	1
	**GAG**	38	1.9	17	2.5	K	AAG	1
	**GAN**	16	0.79	9	1.3			
	**otros**	27	1.3	8	1.2			
**G140**	**GGA**	72	3.6	501	74	S	AGC	7.4
	**GGT**	321	16	11	1.6	S	AGT	1
	**GGC**	1454	72	53	7.8	S	AGC	1
	**GGG**	30	1.5	94	14	S	AGC	3.9
	**GGN**	97	4.8	18	2.6			
	**Others**	42	2.1	3	0.44			
**Q148**	**CAA**	1870	93	601	88	H/R	CAC/CGA	1.8/1
	**CAG**	81	4.0	71	10	H/R	CAC/CGG	5.8/1
	**CAN**	47	2.3	4	0.59			
	**Others**	18	0.9	4	0.59			
**N155**	**AAT**	1848	92	647	95	H	CAT	1.8
	**AAC**	79	3.9	11	1.6	H	CAC	1.8
	**AAN**	38	1.9	7	1.0			
	**Others**	51	2.5	15	2.2			
**G163**	**GGA**	1611	80	321	47	R	AGA	1
	**GGT**	10	0	2	0.29	R	CGT	5.8
	**GGC**	9	0	2	0.29	R	CGC	5.8
	**GGG**	163	8.1	282	41	R	AGG	1
	**GGN**	69	3.4	20	2.9			
	**Others**	154	7.6	53	7.8			
**D232**	**GAT**	1577	78	150	22	N	AAT	1
	**GAC**	213	11	495	73	N	AAC	1
	**Others**	226	11	36	5.3			
**R263**	**AGA**	1810	90	586	86	K	AAA	1
	**AGG**	142	7.0	72	11	K	AAG	1
	**AGN**	54	2.7	16	2.4			
	**Others**	10	0.50	6	0.88			

*AA: Amino acid change which INSTI resistance. † Triplet with the lowest substitution cost according to Theys et al. 2019. ‡SC: Substitution cost (score defined for each triplet according to Theys et al. 2019).

Supplementary Figure 1. Frequency of INSTI-resistance mutations in subtype B and non-B subtype infections. Only mutations with statistically significant differences are shown.

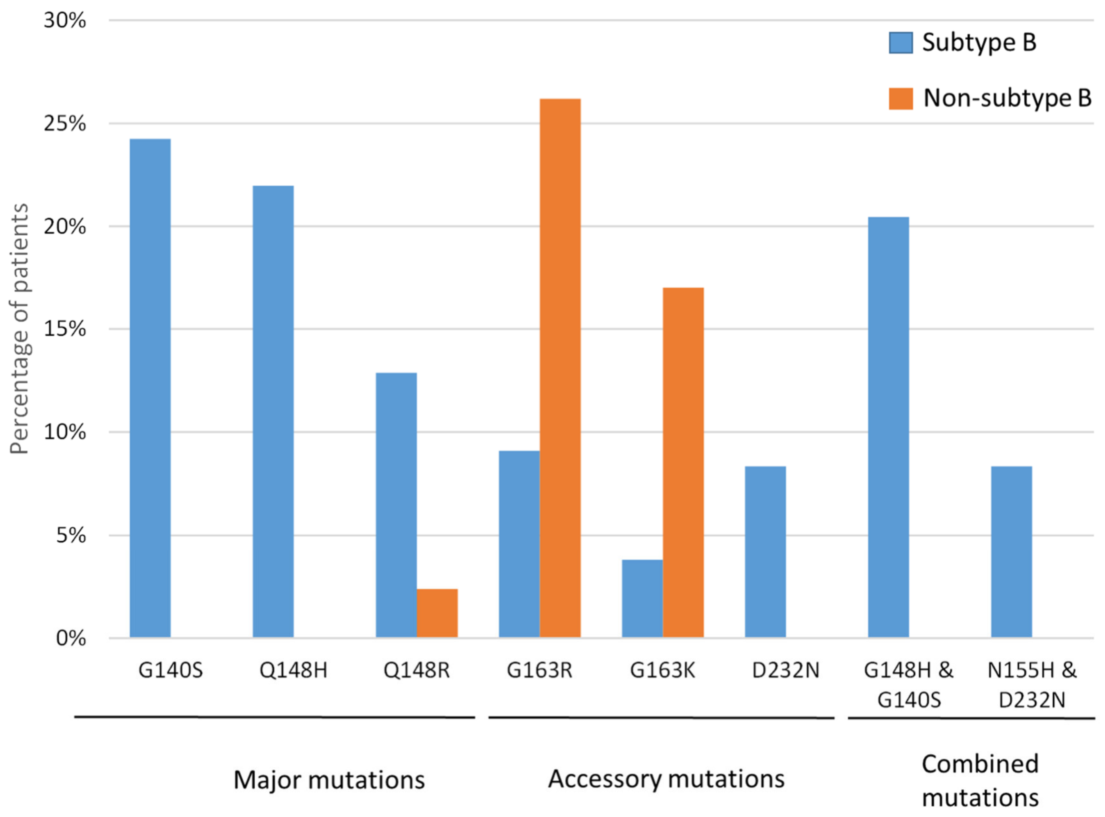

Supplement: Supplementary file 1 [file Presentation_1.pdf]
